# Addressing RNA Integrity to Determine the Impact of Mitochondrial DNA Mutations on Brain Mitochondrial Function with Age

**DOI:** 10.1371/journal.pone.0096940

**Published:** 2014-05-12

**Authors:** Wei Wang, Katja Scheffler, Ying Esbensen, Janne M. Strand, James B. Stewart, Magnar Bjørås, Lars Eide

**Affiliations:** 1 Department of Medical Biochemistry, Oslo University Hospital, University of Oslo, Oslo, Norway; 2 Department of Microbiology, Oslo University Hospital, University of Oslo, Oslo, Norway; 3 Department of Clinical Molecular Biology and Laboratory Sciences (EpiGen), Division of Medicine, Akershus University Hospital and University of Oslo, Lørenskog, Norway; 4 Max Planck Institute for Biology of Ageing, Cologne, Germany; Louisiana State University and A & M College, United States of America

## Abstract

Mitochondrial DNA (mtDNA) mutations can result in mitochondrial dysfunction, but emerging experimental data question the fundamental role of mtDNA mutagenesis in age-associated mitochondrial impairment. The multicopy nature of mtDNA renders the impact of a given mtDNA mutation unpredictable. In this study, we compared mtDNA stability and mtRNA integrity during normal aging. Seven distinct sites in mouse brain mtDNA and corresponding mtRNA were analyzed. Accumulation of mtDNA mutations during aging was highly site-specific. The variation in mutation frequencies overrode the age-mediated increase by more than 100-fold and aging generally did not influence mtDNA mutagenesis. Errors introduced by mtRNA polymerase were also site-dependent and up to two hundred-fold more frequent than mtDNA mutations, and independent of mtDNA mutation frequency. We therefore conclude that mitochondrial transcription fidelity limits the impact of mtDNA mutations.

## Introduction

The mitochondrial theory of aging postulates that mutations in mitochondrial DNA (mtDNA) accumulate with age and result in impaired quality and activity of the mtDNA-encoded proteins. The theory is supported by the fact that mitochondrial function decreases with age, presumably due to accumulation of somatic mtDNA mutations [1]. Mitochondrial dysfunction associated with accumulation of clonal expansions of deletion mutations are reported in nucleoside reverse transcriptase inhibitor (NRTI)-treated individuals [2], [3] and neurodegenerative disorders including Multiple Sclerosis, Alzheimer's Disease and Parkinson's Disease [4]. Although mutated mtDNA are selectively removed during folliculogenesis [5] as well as during maternal transmission [6], inheritable heteroplasmy causes inheritable mitochondrial disease like MELAS when such processes fail [7].

The Polg mutator mouse expresses an error-prone mtDNA polymerase γ and accumulates excessive mtDNA mutations in an age-dependent manner. The Polg mutator evidently demonstrates that mtDNA mutations can result in pathology and shortened lifespan [8]. Although this model has been used to demonstrate the correlation between mtDNA mutations, mitochondrial dysfunction and premature aging, it is questionable to what extent this genetic mitochondrial mutator model represents the molecular mechanisms that underlie mitochondrial dysfunction during normal aging. For instance, the level of mtDNA substitution mutations in old individuals with a corresponding mitochondrial dysfunction does not produce a mitochondrial dysfunction when present in young mutator mice [9]. Rather, mtDNA deletions were suggested to be responsible for age-mediated dysfunction. Accumulation of mtDNA deletions correlated with the phenotype and mutation frequency during normal aging [10]. The deleterious functional effects of deletion mutations are demonstrated clinically in Kearns-Sayre Syndrome [11]. However, genetic models have demonstrated that deletion mutations in up to 60% of the mtDNA molecules are tolerated without manifesting into phenotypic abnormalities [12]. In view of the relatively high tolerance for mtDNA deletion mutations, there is an unexplained discrepancy between the observed mutation frequency during normal aging and the age-associated dysfunction [12]–[14].

The functional impact of mtDNA mutations is hard to predict because of the multiplicity of mtDNA molecules in the cell. mtDNA copy number is also subjected to variations and the heteroplasmic state implies that the likelihood for a mutation to manifest into dysfunctional protein depends on the mtDNA copy number, given that transcription occurs randomly among mtDNA molecules. In view of the redundancy of mtDNA to serve templates for downstream mitochondrial protein components, we reasoned that the best strategy to evaluate mtDNA mutagenesis would be to address the integrity of the mitochondrial RNA (mtRNA). In order for mtDNA mutations to result in functional impairment, the mutations must significantly modify the population of mtRNA molecules. To investigate the impact of mtDNA mutations with age, we developed an assay to determine mtRNA integrity with high resolution and used this technology to compare mtDNA mutagenesis and mtRNA error frequency in brains from young mice with those from old mice, which were associated with impaired mitochondrial function. Our results show that mtRNA error frequency can be used to validate mtDNA mutagenesis.

## Materials and Methods

### Ethics statements

The project was approved by the Norwegian Animal Research Authority and in accordance with the laws and regulations controlling experimental procedures in Norway and the European Union's Directive 86/609/EEC.

### Animal and Tissue Collection

Wild-type C57BL/6 mice (males/females) were either bred in-house (old group, 18 months, n = 8) or purchased from Taconic (young group, 1 month, n = 8). Heterozygous (Polg^mut/+^, n = 3) and homozygous (Polg^mut/mut^, n = 3) mutator mice were about 9 months old (36–39 weeks). The project was approved by the Norwegian Animal Research Authority and in accordance with the laws and regulations controlling experimental procedures in Norway and the European Union's Directive 86/609/EEC. Mouse whole brain without cerebellum was collected immediately after the animals were sacrificed. One of the hemispheres was submerged in RNAlater solution (Sigma) for DNA/RNA extraction and the other used for detection of mitochondrial functions.

### Isolation and Quantification of Nucleic Acids

The RNAlater-stored brain tissues were homogenized with FastPrep-24 (MP Biomedical, USA). Total DNA was isolated by the DNeasy Blood and tissue kit (Qiagen). Total RNA was isolated using the RNeasy Mini Kit (Qiagen) according to manufacturer's protocols with modifications. In brief, 20–30 mg of homogenized brain tissue was lysed and applied to spin column. After the column was washed with AW1 buffer, DNase I (Qiagen) was added to the column and incubated at room temperature for 15 min to remove residual DNA. To completely remove possible contamination of the residual DNA, eluted RNA was further treated with rDNase I at 37 °C for 20 min according to manufacturer's protocol (DNase treatment & removal, Ambion). Quantification of DNA and RNA was done on Nanodrop spectrophotometer (Thermo Scientific).

### Analysis of mtDNA Point Mutation Frequency

Double stranded mtDNA mutation frequency was estimated as described previously with modifications [15]. Briefly, total DNA was treated with S1 nuclease (Qiagen; 10 U) for 15 min at 37 °C and subsequently digested with TaqI restriction enzyme (New England Biolabs; 100 U) at 65 °C for 1 h to remove single strands, damaged DNA and non-mutated DNA. The remaining mutated TaqI restriction sites were quantified in a qPCR reaction using the specific primers listed in Table S1 in File S1. To ensure complete digestion, an additional TaqI treatment (100 U; 65 °C for 15 min) was performed on the digested DNA samples prior to the subsequent qPCR analyses. Complete digestion was verified by subsequent TaqI treatment of qPCR products. Mutation frequency is found as (2exp(CT^TaqI –^ CT ^NT^) * 4)^−1^ per nt. The presence of nuclear mtDNA fragments (numts) [16] could potentially interfere with our data as we analyze total DNA and pseudogenes with altered nucleotide sequence potentially could generate positive signals in our restriction cleavage inhibition-based assay. However, the melt-curve analyses indicated that we always amplified the same PCR fragment. The CT values for the seven mtDNA sites were in the same range and thus supports that do not interfere with our results.

### Analysis of mtRNA Errors

cDNA was prepared from 0.5–1.0 µg total RNA using the High Capacity cDNA reverse transcription kit (Applied Biosystems). Subsequently, in order to create double-stranded cDNA for TaqI digestion, PCR was performed with 100 ng of cDNA with a HotStart PCR system (5 Prime) in a total volume of 50 µl containing 1 × HotStart PCR buffer, 0.2 mM of dNTPs, 0.2 µM of each specific forward and reverse primer and 2.5 U of Taq DNA polymerase. The PCR reactions were performed for 10 cycles. The amplified products were diluted to give appropriate initial input for qPCR quantification. Then 100 ng of DNA were either left untreated (nt) or treated with TaqI (TaqI; 100 U, 65 °C for 1 h). The mtRNA frequency was calculated from the resulting CT values using the same formula as for mtDNA mutations. The procedure is outlined in Figure S1.

The mtRNA deletions were evaluated by analyzing possible truncated products in a PCR amplification of extended cDNA regions. cDNA was first amplified using primers 12S-F6 (Table S1 in File S1) (full-length product: 685 bp trancripts) of 12S ribosomal RNA with a HotStart PCR for 19 cycles in order to obtain appropriate amounts of DNA, whereupon an aliquote of 100 ng was digested with TaqI enzyme (100 U, 65°C for 1 h). 6 ng of digested or non-digested samples were further used as templates to amplify two amplicons of 206 and 442 bp with primers 12S and 12S-F4 in a 40-cycle PCR reaction, respectively (Table S1 in File S1). The amplified products were analyzed by Agilent 2100 Bioanalyzer using Agilent DNA 1000 regents (G2938-80020, Agilent Technologies).

### Mitochondrial Gene Expression

Mitochondrial gene expression analyses were performed by qPCR with the primer sets provided in Table S1 in File S1. Total RNA was isolated and reverse-transcribed into cDNA and quantified by qPCR. Expression levels were determined from the difference in ΔCT values. The housekeeping gene *Gapdh* was used as internal control.

### Western Analysis

Mitochondrial proteins (5–10 µg) were separated on a 12% NuPAGE Bis-Tris gel (Invitrogen) and blotted onto PVDF membranes (Millipore). After blocking in 5% dry milk in TBST buffer (50 mM Tris pH 7.4, 150 mM NaCl, 0.1% Tween 20) for 1 h at room temperature, blots were probed for specific subunits of the respiratory complexes overnight at 4°C. Primary antibodies used were: anti-total OXPHOS antibody cocktail (1∶2000, MitoSciences), anti-ND6 (1∶400, Biorbyt) and as loading control anti-VDAC (1∶500, Abcam). As secondary antibodies alkaline phosphatase (AP)-conjugated goat anti-rabbit IgG (1∶30000) and goat anti-mouse IgG (1∶5000) from Santa Cruz Biotechnology were used. Fluorescent signals from enhanced chemifluorescence substrate (GE Healthcare) were detected by Bio-Rad ChemiDoc XRS+ and analyzed with Image Lab Software (Bio-Rad).

### Statistical Analysis

Pearson correlation was used to evaluate correlations between DNA and RNA and SPSS version 10 was used for the statistical analysis. Wilcoxon signed-rank test was employed to evaluate effect of age on mutagenesis and RNA integrity. Student's t-test with two-tailed distribution and equal variance was used to calculate significance, *p<0.05; **p<0.01.

## Results

### mtDNA Mutation Frequency Varies More Between Specific Sites Than With Age

We used a qPCR-based method for high-resolution quantification of mtDNA mutations in TaqI restriction sites that was validated and described previously [15]. Total DNA from the hemispheres was used for the analyses rather than DNA from an isolated subfraction of brain mitochondria. We selected seven different sites in the mitochondrial genome for assessment of mutations in mtDNA during aging. We chose mice of 1 and 18 months age to represent young and old animals, respectively. Although the age of 1 month does not represent all aspects of a developed mouse brain, we selected this age to limit putative contribution of age on somatic mutagenesis. 18 months was likewise chosen to avoid putative age-associated disease-driven mtDNA mutagenesis from influencing the results. We confirmed that brain mitochondrial function was impaired in the old cohort (about 35% reduction in complex I, II and V; Figure S1).

**Figure 1:**
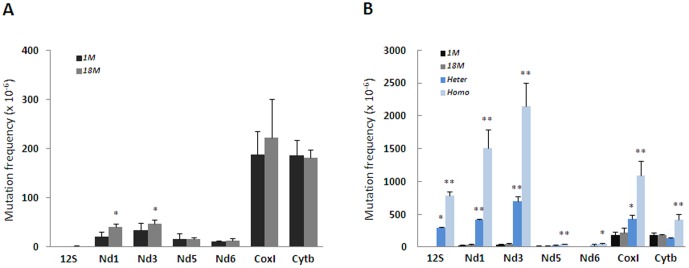
Site- and Age- Dependent Accumulation of Mutations in Brain mtDNA. (A) DNA from young (1 month, n = 8) and old (18 months, n = 8) mice were analyzed for mutation rate in 7 different sites in the mtDNA. DNA was isolated from whole brain. (B) DNA from heterozygous (mut/+, n = 3) and homozygous (mut/mut, n = 3) mutator mice (9 months) were analyzed in the same sites. Figures show mean with SD. **p<0.01, *p< 0.05.

The mtDNA mutation frequencies in brains from young individuals varied remarkably between the different loci, from 1.3 × 10^−6^ per nt in the 12S ribosomal RNA to 188 × 10^−6^ per nt in the CoxI site (Figure 1A). Mitochondrial DNA from aged brains (18 months) displayed significantly elevated mutagenesis in the Nd3 (+ 40%) and Nd1 (+ 90%) sites and borderline significant (p = 0.07) increase in the 12S site (+ 50%), whereas the Nd5, Nd6, Cytb and CoxI sites were unaffected. Mutations did not generally increased with age as implied from the Wilcoxon signed-rank test. In line with the common responsiveness of the Nd1 and Nd3 sites to age, we observed a significant correlation between these (r = 0.58) among the individuals. A significant correlation was identified between 12S and Nd3 (r = 0.61) and 12S and Nd6 (r = 0.744) as well. These data suggest that increased mtDNA mutagenesis during age is region dependent. We included DNA from Polg mutator mice that express one or two alleles of the 3′-5′ exonuclease-deficient DNA polymerase γ as controls. The mutator mice accumulate several hundredfold more mutations than control mice with age [8]. This was confirmed by us as mtDNA from the Polg mutator mice (9 months age) contained up to 400-fold higher levels of mutations than control mice, dependent on the site and allelic dose (Figure 1B). There was no apparent correlation between the site-specific mutation level and the effect of 5′ exonuclease-deficiency. However, the 12S, Nd1 and Nd3 sites, which were relatively most affected in the Polg mutator mice (40 to 400-fold versus <5-fold for the remaining) were those that increased significantly with age.

### Site-Specific Transcriptional Errors are Independent of Age and Mutation Frequency

**Figure 2:**
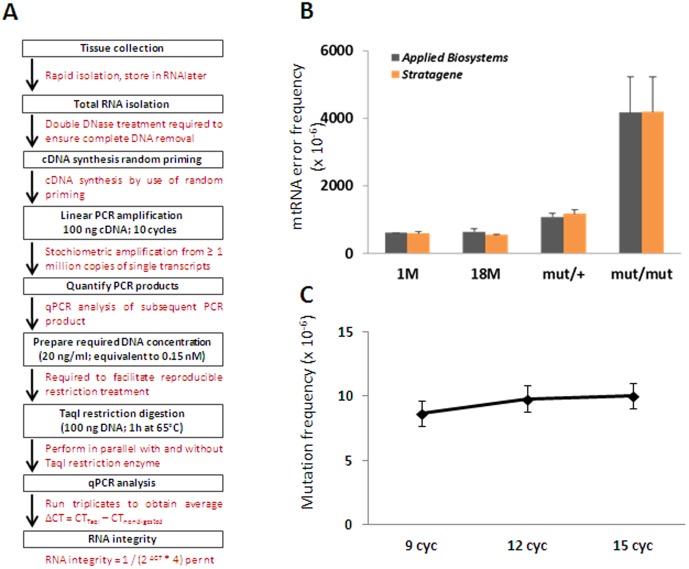
Validation of Method. (A) Flow chart illustrating the procedure for detection of mtRNA integrity. (B) Comparison of RNA integrity in CoxI site obtained by using two different commercially available reverse transcriptases. (C) Estimation of errors introduced in the PCR amplication. The 12S rRNA region was amplified with increasing cycle number and 100 ng PCR product was either digested with TaqI or left untreated and subsequently analyzed for mutations. The mutation frequency is plotted as function of PCR cycle number. The figures show mean with SD from three independent experiments.

**Figure 3:**
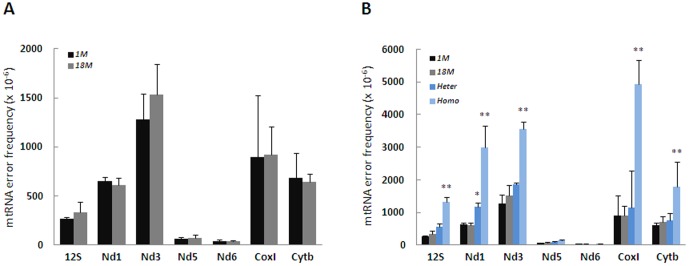
RNA Error Frequency is Age-Independent in Controls but Elevated in Mutator Mice. (A) mtRNA errors were measured as described at the same 7 loci as for mtDNA from brains of young (1 months, n = 8) and old (18 months, n = 8). (B) mtRNA errors in heterozygous (mut/+, n = 3) and homozygous (mut/mut, n = 3) mice. Figures show mean with SD. p**<0.01 vs. 18 months, p*< 0.05 vs. 18 months.

The large variations in mutation frequency suggested that the penetrance might be site-specific. Since there are multiple copies of mtDNA, the frequency of mutations must be higher than the rate of ribonucleotide misincorporation by mitochondrial RNA polymerase at the specific site in order to manifest into mitochondrial dysfunction. We developed a high resolution assay to analyze mtRNA errors introduced in the same TaqI restriction recognition sequences. The procedure is illustrated in Figure 2A. Total RNA was isolated from the same samples as used for DNA isolations, and used in reverse transcriptase reactions to produce cDNA that was subsequently amplified by PCR and finally analyzed for TaqI-resistant sequences. To control for the artificial errors introduced by this procedure, we compared misincorporation of two high fidelity reverse transcriptases and estimated incorporation errors by the DNA polymerase in the subsequent PCR amplification. RNA from the mutator mouse served as an ultimate control for RNA errors that manifest from mutations in mtDNA. Two commercially available high fidelity reverse transcriptases (with a declared fidelity of 2 to 7 × 10^−6^ per nt according to the manufacturer) gave identical error rates in the CoxI site for the different genotypes, demonstrating that the fidelity of reverse transcriptases is not restricting the conclusions (Figure 2B). The 12S and Nd1 sites were additionally tested by the two reverse transcriptases and gave similar conclusion (data not shown). Furthermore, by amplifying DNA and subsequently quantifying mutated PCR products, we determined that the errors introduced by 6 cycles of PCR (from 9 to 15) yielded less than 2 × 10^−6^ per nt (Figure 2C). Together, the procedure therefore generates artificial errors with an estimated frequency of less than 1 × 10^−5^ per nt. When this procedure was applied on samples from young and old mice, we found that the frequencies of misincorporated ribonucleotides in mtRNA *in vivo* were at least 3-fold higher than this and varied in a site-specific manner (Figure 3). Overall, there was a 35-fold variation in error frequency. Similarly as for mtDNA mutation frequency, we used Wilcoxon signed-rank test on relative average mtRNA error levels and found that the two age groups were not significantly different (p>0.05). The mtRNA error frequency spans from 0.3 to 15 × 10^−4^ per nt, and overrides site-specific mtDNA mutation frequency from 3 to 200-fold (Figure 3A). We did not find any correlation between mtRNA error frequencies and mtDNA mutations, except for an interesting pattern in the Nd5/Nd6 sites where the mutation frequency in Nd6 displayed a negative correlation with mtRNA integrity in the neighboring Nd5 site (r = −0.59, p = 0.021). The lack of positive correlation between mutations and mtRNA errors strongly indicates that the mtDNA mutation frequency at a given site does not predict the corresponding mtRNA error frequency. Only mtDNA from the Polg mutator mice contained mutations at a level that possibly could influence the mtRNA integrity (Figure 1B). This was verified by Pearson correlation analyses, which demonstrated strong correlation (r>0.84) between mtDNA mutation and mtRNA errors in the 12S, Nd1, Nd3, Cytb and CoxI sites in the mutator mice, thereby demonstrating the penetrance of the mutator genotype. The homozygous mutator mice had in average 2.6-fold more mtRNA errors than wt. In contrast, the mtRNA error frequencies in the heterozygous mutator mice were not significantly different from wt, except for the Nd1 site (Figure 3B). The relatively low effect of single allele *PolG* mutation on mtRNA errors in heterozygous mutator mice correlates with its innocuous phenotype compared to homozygous mutator mouse [8].

Since the relatively low mtRNA error frequency determined for the Nd6 site is in the range of the estimated resolution of the method, it was a possibility that the Nd6 mtRNA error frequency was overestimated. As an internal control of the resolution of the assay, we addressed the frequency of errors in nuclear ribosomal RNA by the same procedure. However, in contrast to mtRNA, we were not able to quantify the exact number of TaqI-resistant cDNA from the nuclear 28S ribosomal RNA because the amount of potential templates was under the detection limit. However, based on the amount of non-digested molecules and this limit, we estimated an upper range of the errors in the 28S rRNA to be 2 × 10^−5^ per nt (Figure S2). The high fidelity of 28S rRNA transcription is expected because of the strong proofreading activity of RNA polymerase I [17] Hence, these results show that the resolution of the procedure is sufficiently high to detect mtRNA errors but not rRNA errors, and demonstrates that the lowest error frequency in the Nd6 site is 3.2 × 10^−5^ per nt.

### No Indications of mtRNA Alterations with Age

**Figure 4:**
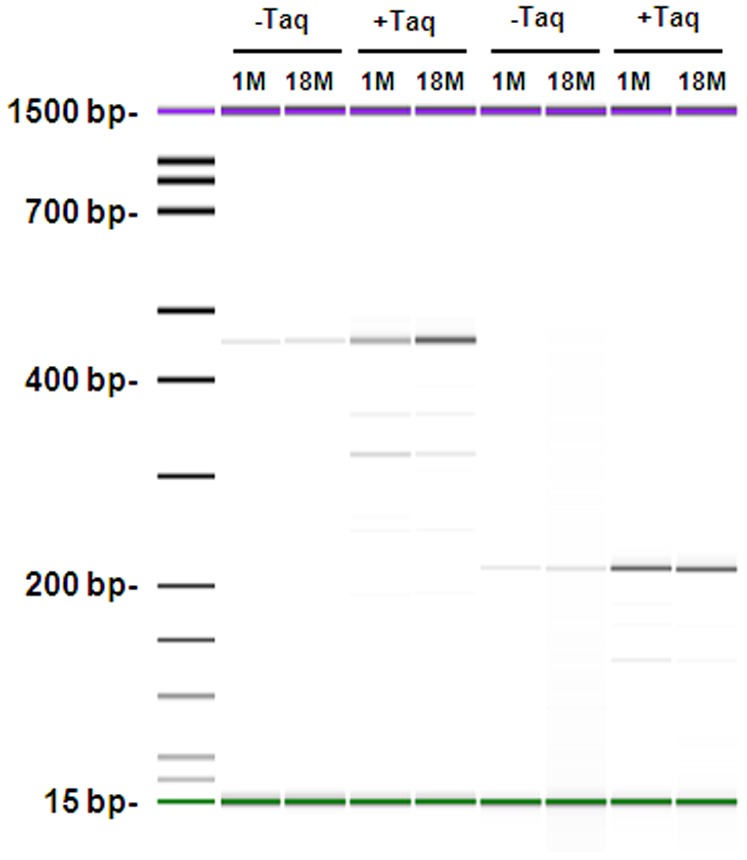
Level of RNA Deletions is Age-Independent. Intentional capture of mtRNA deletions by PCR amplification of extended cDNA regions.

**Figure 5:**
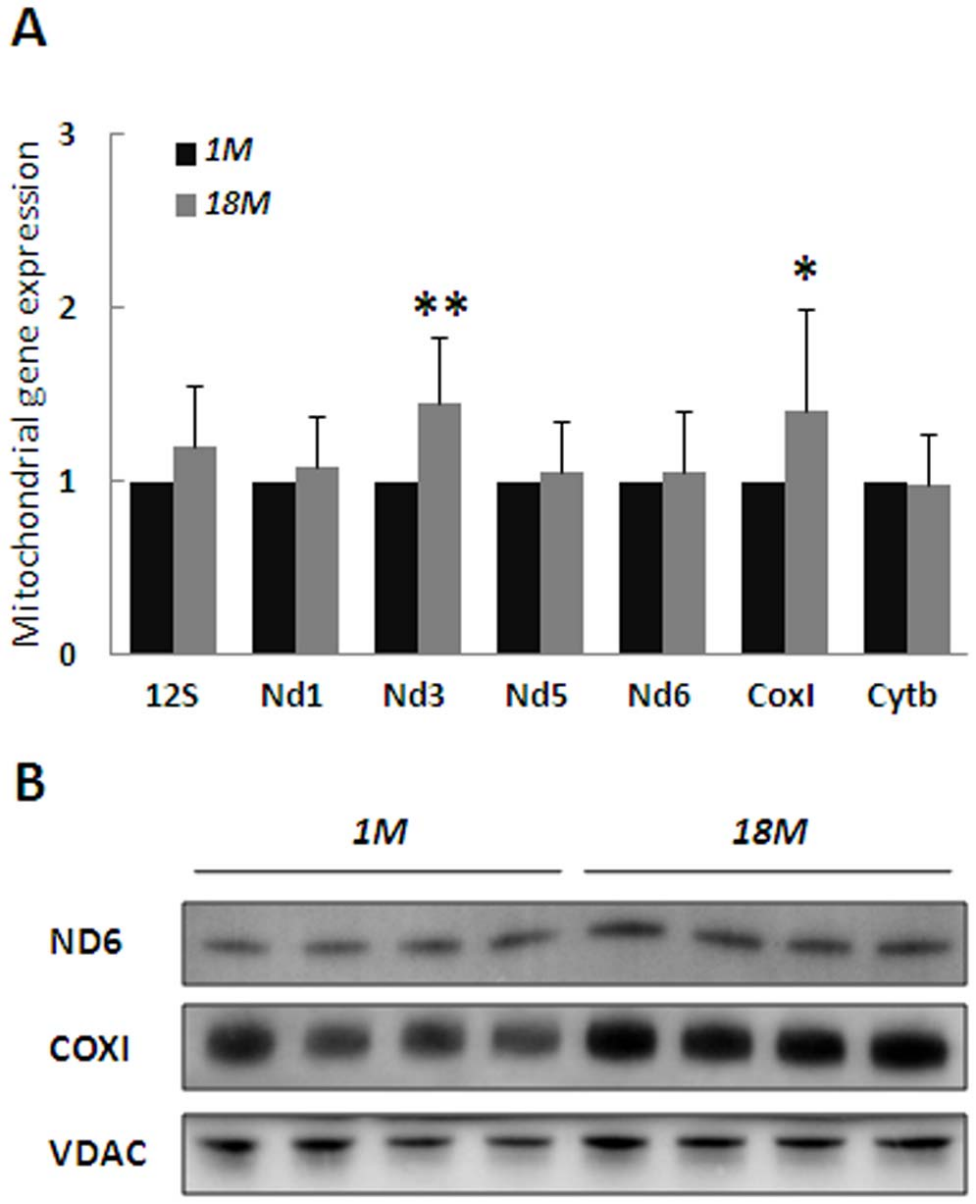
Stable Expression of Genes and Proteins with Age. (A) Expression of mitochondrial genes was analyzed by RT-qPCR normalized to *Gapdh* and presented relative to 1 month old mice. (B) Expression levels of proteins were evaluated by western analyses using antibodies as specified in Experimental Procedures. The relative increase in COXI and ND6 with age is indicated. Figure shows mean with SD. **p<0.01, *p< 0.05.

We evaluated the impact of mtDNA deletion mutations by an analogue strategy. The contribution of mtDNA deletions to errors in mtRNA was determined by intentional capture of mtRNA deletions by PCR amplification of extended cDNA regions. We did not identify detectable deleted transcripts in the template mixture by this approach (Figure 4). After removing intact cDNA by TaqI digestion prior to PCR capturing, we observed truncated PCR products from both age groups. However, despite the preference for truncated products to be amplified in PCR, full-length PCR products were still more abundant in both young and old brain. We conclude that deletions contribute less than single base substitutions to the TaqI resistance (Figure 4). The innocuous impact of age on mtRNA integrity was underlined by the normal or increased mitochondrial gene expression in the old group (Figure 5A), which correlated with elevated expression of mitochondrial proteins in the older individuals (Figure 5B).

## Discussion

Here, we show that subtle increases in mtDNA mutation frequency during normal aging are insufficient to influence the quality of mtRNA in seven selected sites. The mtRNA integrity is determined by the fidelity of the mtRNA polymerase, which masks mtDNA mutation frequency by an order that is dependent on the site. The mutator mice lacking the 3′-5′ exonuclease accumulate mutations at a frequency that is in range of the mtRNA polymerase misincorporation. Consequently, we only observe positive correlations between mtDNA mutagenesis and mtRNA integrity in these mutator mice.

Our results are in line with deep-sequencing analysis of liver mtDNA [18] that questions the general effect of aging on mtDNA mutagenesis. As we have focused on seven 4-base sequences, we cannot rule out that mutations outside these sites contribute to the age-associated mitochondrial dysfunction. Our data nevertheless indicate that some sites are more prone to mutagenesis with age, and may correlate with those that undergo progressive clonal expansion to preclude mitochondrial dysfunction [2], [4], [19]. Our method can be used to identify the potential of a position to undergo clonal expansion. Carriers of the common deletion mutation may have up to 60% of mutated mtDNA molecules without manifesting into disease [12]. It would be interesting to address mtRNA integrity in carriers of the common deletion, to correlate this with the onset of disease, known as the Kearns–Sayre syndrome.

In this study, we focused on brain mtDNA mutagenesis. Brain and heart were previously shown to accumulate mitochondrial mutations to a similar extent with age [9]. We previously detected equal mutation frequencies in the same sites in liver and brain, but higher in lung [15]. As a rough estimate, the average brain mtDNA mutation frequency in all seven sites is 0.7 × 10^-4^ per nt, which is in coherence with results obtained by the deep-sequencing analysis of liver mtDNA [18]. Recent ultra-sensitive sequencing of human brain mitochondria has shown that age indeed increase mutagenesis. The apparent contradiction to our data could be that the relative age of the mice in our cohort was relatively much lower than the human cohort in the cited reference [20]. In contrast to mouse brain, the frequency of mtDNA mutations in liver did not change with age in any sites and suggests that age influences mtDNA mutagenesis in a tissue/cell type-specific manner. In particular, this is demonstrated by the reduction in mtDNA mutation level in colorectal cancer [21].

Interesting age-independent relationships between the mutation frequencies within individuals were discovered. We found a positive correlation between Nd1 and Nd3, which might be expected as both sites showed age-associated mutagenesis. However, a stronger correlation was observed between mutation frequencies in 12S and Nd1, as well as in 12S and Nd6, which was independent of age. In comparison, none of the other sites (Nd5, Cytb, CoxI) seemed to be interconnected. Perhaps a subset of mtDNA genes/sequences is particularly important in subregions of the brains, and that the quality of these regions is selected for. An analogy to this is observed during the purifying deselection of non-synonymous mutations in the protein-coding mtDNA genes during transmission [6], suggesting that mtDNA mutagenesis is under functional control. An analogue indication of a targeted mutagenesis was suggested in very young human brain samples as well [20].

The presence of nuclear mtDNA fragments (numts) [16] could theoretically introduce errors as we analyze total DNA and pseudogenes with altered nucleotide sequence potentially could generate positive signals in our restriction cleavage inhibition-based assay. However, since the primers used in the qPCR display similar efficiency and the CT values are comparable for the seven sites, it means that the mtDNA copy number is identical independent of the site to quantify. Again, this demonstrates that numts do not contribute significantly to the readout in any of the seven sites. Additionally, the melt-curve analyses do not indicate the presence of another amplicon to be amplied. Nevertheless, only the numts that deviate from mtDNA sequences would be detected, thus the potential outcome of numts on our conclusion would then be that the real mtDNA mutation rates are lower than we report. The lack of correlation between mutation frequency and mtRNA error frequency makes it very unlikely that numts are sufficiently frequent to interfere with our results.

The expression of mitochondrial genes in the old cohort was not reduced, supporting our findings that mtRNA integrity was unaffected by aging as substitutions and deletions are likely to impair the stability of mtRNA. In view of our data we therefore believe that the correlation between mtDNA deletion and mitochondrial dysfunction with age is merely a secondary effect with minor functional impacts. In fact, the general expression of mitochondrial transcripts was slightly increased in old mice, presumably as an adaptive response to the observed mitochondrial function impairment. The innocuous impact of mtDNA mutations on age-related mitochondrial dysfunction is supported by the parallel reduction in complex II, which is entirely nuclear encoded (Figure S1).

In summary, addressing mtRNA error frequency to evaluate mtDNA mutagenesis is a vital approach to determine the impact of mitochondrial mutations and can be used to test putative contribution of mtDNA mutations in various diseases.

## Supporting Information

File S1 **This Supporting Information file combines Supporting Methods and Table S1.** Figure S1 and S2 are provided as separate files.
(DOCX)

Figure S1 **Mitochondrial dysfunction with age.** Enzymatic activity measurements of complex I, II and V in isolated brain mitochondria reveal an average 35% reduction in old (18 months, n = 11) compared to young mice (1 month, n = 4). Figure shows mean with SD, p**< 0.01.
(PDF)

Figure S2 **Overestimate of 28S rRNA error frequency.** Errors in nuclear 28S ribosomal RNA were estimated with a slightly modified procedure. RNA was reverse transcribed, and the cDNA/RNA hybrid was treated with 100 units of TaqI for 1 h at 65°C and followed by qPCR analysis. Figure shows mean with SD.
(PDF)
