# Supplementary material for: Addressing RNA Integrity to Determine the Impact of Mitochondrial DNA Mutations on Brain Mitochondrial Function with Age
Source: PLoS One. 2014 May 12;9(5):e96940. doi: 10.1371/journal.pone.0096940 (PMC4018447; doi:10.1371/journal.pone.0096940)
Supplement: File S1 — This Supporting Information file combines Supporting Methods and Table S1. Figure S1 and S2 are provided as separate files. (DOCX) [file pone.0096940.s001.docx]

SUPPORTING INFORMATION

**Supporting methods**

**Mitochondrial Preparation and Complex Analyses**

Mitochondria from freshly prepared brain hemispheres were isolated as described previously [1]. Mitochondrial proteins were adjusted to 40 µg and enzymatic activities were measured in duplicates using a 96 well plate reader (Wallac 1420 Multilabel Counter). Complex I, ATPase activity, representing complex V and citrate synthase activity were assessed as described [2]. For complex II activity, isolated mitochondria were incubated in a reaction mixture containing 10 mM potassium phosphate buffer (pH 7.4), 2 mM EDTA, 1 mg/ml BSA, 0.2 mM ATP, 4 µM rotenone, 80 µM dichlorophenolindophenol and 10 mM succinate for 10 min at 30°C. The reaction was started by adding 0.135 mM decylubiquinone and enzymatic activity was determined by following absorbance at 600 nm every 30 sec for 5 min. The complex activities were presented relative to citrate synthase activity.

**Supporting Table S1** Oligonucleotides used in the study

| Loci | Amplicon (nt) | Sequence (5’-3’) |
| --- | --- | --- |
| 12S | 206 | actcaaaggacttggcggta  agcccatttcttcccatttc |
| 12S-F4 | 442 | cacgacagctaagacccaaa  cggtgtgtgcgtacttcatt |
| 12S-F6 | 682 | cacgggactcagcagtgata  cggtgtgtgcgtacttcatt |
| Nd1 | 133 | ttacttctgccagcctgacc  cggctgcgtattctacgtta |
| Nd3 | 82 | gcattctgactcccccaaat  gacgtgcagagcttgtaggg |
| Nd5 | 111 | tcagacccaaacatcaatcg  cccttctcagccaatgaaaa |
| Nd6 | 154 | aacaaccaaccaaaaaggctta  gctgggtgatctttgtttgc |
| CoxI | 117 | ctgagcgggaatagtgggta  aaagcatgggcagttacgat |
| Cytb | 120 | cagccttttcatcagtaacaca  ctcgtccgacatgaaggaat |

The primers were designed with Primer 3 (<http://frodo.wi.mit.edu/>) except for *12S* ribosomal RNA gene which was adapted from previous report [3]. Based on TaqI restriction sites in mtDNA genome, seven pairs of primers were chosen to screen different genes. All primer sets were optimized with mtDNA and mtRNA to give CT values within a reliable range.

**References**

[1] R.Halsne, Y.Esbensen, W.Wang, K.Scheffler, R.Suganthan, M.Bjoras, L.Eide. Lack of the DNA glycosylases MYH and OGG1 in the cancer prone double mutant mouse does not increase mitochondrial DNA mutagenesis, DNA Repair (Amst) 11 (2012) 278-285.

[2] A.Barrientos, F.Fontanesi, F.Diaz. Evaluation of the mitochondrial respiratory chain and oxidative phosphorylation system using polarography and spectrophotometric enzyme assays, Curr. Protoc. Hum. Genet. Chapter 19 (2009) Unit19.

[3] M.Vermulst, J.H.Bielas, G.C.Kujoth, W.C.Ladiges, P.S.Rabinovitch, T.A.Prolla, L.A.Loeb. Mitochondrial point mutations do not limit the natural lifespan of mice, Nat. Genet. 39 (2007) 540-543.
