# Supplementary material for: Addressing RNA Integrity to Determine the Impact of Mitochondrial DNA Mutations on Brain Mitochondrial Function with Age
Source: PLoS One. 2014 May 12;9(5):e96940. doi: 10.1371/journal.pone.0096940 (PMC4018447; doi:10.1371/journal.pone.0096940)
Supplement: Figure S1 — Mitochondrial dysfunction with age. Enzymatic activity measurements of complex I, II and V in isolated brain mitochondria reveal an average 35% reduction in old (18 months, n = 11) compared to young mice (1 month, n = 4). Figure shows mean with SD, p**< 0.01. (PDF) [file pone.0096940.s002.pdf]

Supporting Figure S1

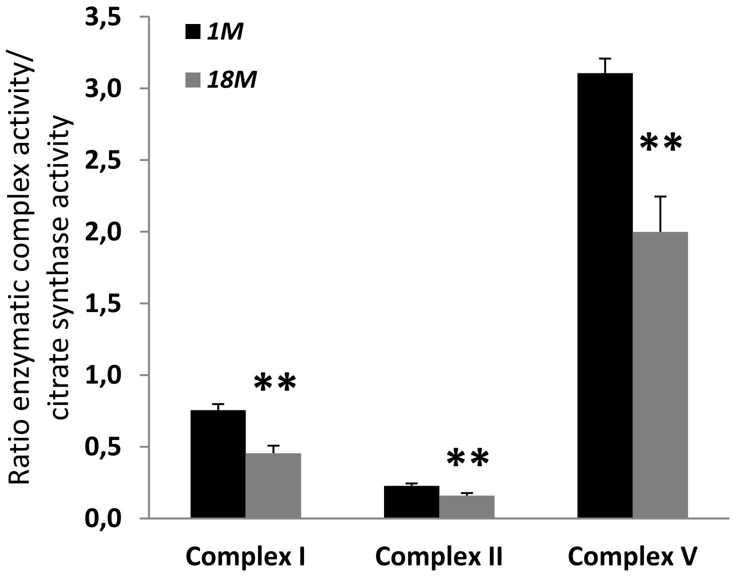

Mitochondrial dysfunction with age. Enzymatic activity measurements of complex I, II and V in isolated brain mitochondria reveal an average 35 % reduction in old (18 months, n=11) compared to young mice (1 month, n=4). Figure shows mean with SD, p**< 0.01
