# Supplementary material for: Addressing RNA Integrity to Determine the Impact of Mitochondrial DNA Mutations on Brain Mitochondrial Function with Age
Source: PLoS One. 2014 May 12;9(5):e96940. doi: 10.1371/journal.pone.0096940 (PMC4018447; doi:10.1371/journal.pone.0096940)
Supplement: Figure S2 — Overestimate of 28S rRNA error frequency. Errors in nuclear 28S ribosomal RNA were estimated with a slightly modified procedure. RNA was reverse transcribed, and the cDNA/RNA hybrid was treated with 100 units of TaqI for 1 h at 65°C and followed by qPCR analysis. Figure shows mean with SD. (PDF) [file pone.0096940.s003.pdf]

Supporting Figure S2

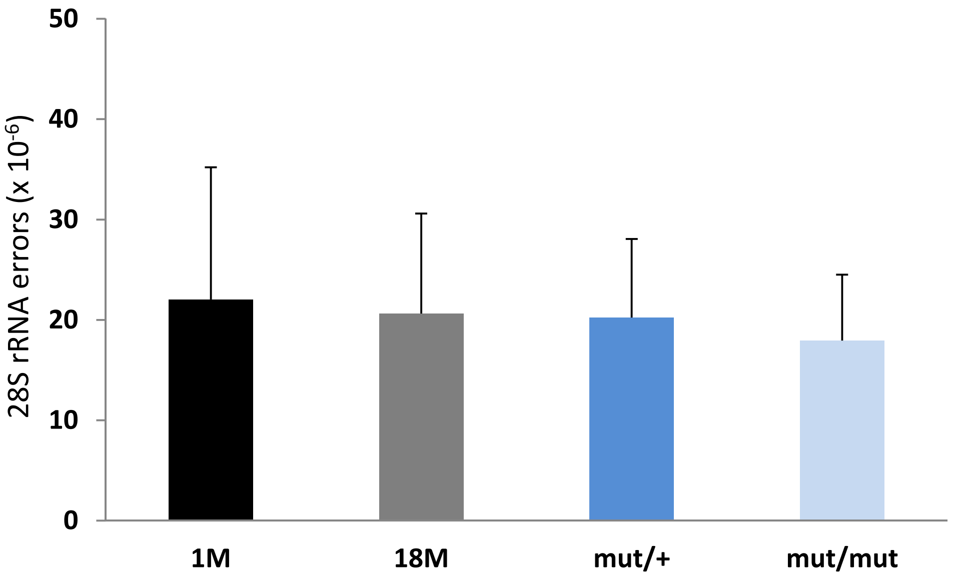

Overestimate of 28S rRNA error frequency. Errors in nuclear 28S ribosomal RNA were estimated with a slightly modified procedure. RNA was reverse transcribed, and the cDNA/RNA hybrid was treated with 100 units of TaqI for 1 h at 65˚C and followed by qPCR analysis. Figure shows mean with SD.
